# Effects of Light, Food Availability and Temperature Stress on the Function of Photosystem II and Photosystem I of Coral Symbionts

**DOI:** 10.1371/journal.pone.0030167

**Published:** 2012-01-13

**Authors:** Mia O. Hoogenboom, Douglas A. Campbell, Eric Beraud, Katrina DeZeeuw, Christine Ferrier-Pagès

**Affiliations:** 1 Ecophysiology and Ecology Research Team, Centre Scientifique de Monaco, Monaco, Monaco; 2 Biology Department, Mount Allison University, Sackville, New Brunswick, Canada; USDA-ARS, United States of America

## Abstract

**Background:**

Reef corals are heterotrophic coelenterates that achieve high productivity through their photosynthetic dinoflagellate symbionts. Excessive seawater temperature destabilises this symbiosis and causes corals to “bleach,” lowering their photosynthetic capacity. Bleaching poses a serious threat to the persistence of coral reefs on a global scale. Despite expanding research on the causes of bleaching, the mechanisms remain a subject of debate.

**Methodology/Principal Findings:**

This study determined how light and food availability modulate the effects of temperature stress on photosynthesis in two reef coral species. We quantified the activities of Photosystem II, Photosystem I and whole chain electron transport under combinations of normal and stressful growth temperatures, moderate and high light levels and the presence or absence of feeding of the coral hosts. Our results show that PS1 function is comparatively robust against temperature stress in both species, whereas PS2 and whole chain electron transport are susceptible to temperature stress. In the symbiotic dinoflagellates of *Stylophora pistillata* the contents of chlorophyll and major photosynthetic complexes were primarily affected by food availability. In *Turbinaria reniformis* growth temperature was the dominant influence on the contents of the photosynthetic complexes. In both species feeding the host significantly protected photosynthetic function from high temperature stress.

**Conclusions/Significance:**

Our findings support the photoinhibition model of coral bleaching and demonstrate that PS1 is not a major site for thermal damage during bleaching events. Feeding mitigates bleaching in two scleractinian corals, so that reef responses to temperature stresses will likely be influenced by the coinciding availabilities of prey for the host.

## Introduction

Coral bleaching is a global issue that threatens the persistence of coral reefs, the world's most diverse marine ecosystems [1], [2], [3]. Over the past 15 years, several major bleaching events have occurred around the globe and caused widespread mortality of corals [4]. Consequently, coral cover on reefs throughout the Indo-Pacific region is declining at a rate of approximately 3,100 km^2^ per year due to bleaching and other anthropogenic impacts [5], [6]. Bleaching events are caused by anomalously high sea surface temperatures that exceed the thermal thresholds for corals and their symbionts - photosynthetic dinoflagellates from the genus *Symbiodinium* commonly known as zooxanthellae. In the presence of moderate to high light intensities, excessive temperature damages the photosynthetic machinery of the zooxanthellae, leading to a decrease in the size and/or pigmentation of the symbiont population and causing the characteristic white colour of bleached corals [7]. The specific mechanisms underlying this process remain a subject of debate.

Temperature-induced coral bleaching is thought to result from the impairment of photosystem 2 (PS2) function, due to accumulated light-dependent damage to a key protein (D1) found in PS2 reaction centres [8], [9], [10]. Nevertheless, several other components of the photosynthetic machinery are also sensitive to temperature and may contribute to the onset of bleaching, or exacerbate the effects of lower PS2 function once bleaching begins. For instance, excessive temperatures can inactivate Rubisco [11], [12] and interrupt the reactions of the Calvin Cycle [13]. The integrity of thylakoid membranes is also compromised at high temperatures leading to an impairment of ATP production [14], [15]. Furthermore, synthesis of the protein components of the light-harvesting antennae declines at elevated temperatures [16] further lowering the overall capacity for photosynthesis. Despite this increasing understanding of the effects of temperature stress on the coral symbiont photosynthetic machinery, the sensitivity of symbiont photosystem 1 (PS1) to temperature and other stressors remains largely unknown.

Much of our understanding of the mechanisms of coral bleaching comes from studies using pulse-amplitude-modulated (PAM) fluorometry techniques [13] to measure photosynthetic activity based on the variable fluorescence signal returned from PS2 during illumination with a light pulse of sufficient intensity to saturate all PS2 reaction centres [17], [18]. Early studies using submersible PAM devices were instrumental in identifying changes in PS2 function due to seasonal changes in temperature [19] and depth related changes in light [20], [21]. More recently, fluorescence techniques have revealed that the oxygen evolving complex, which catalyses the splitting of water into protons and O_2_ during the light-reactions of photosynthesis, is relatively insensitive to thermal stress [22]. Although the activity of PS2 can be monitored through changes in fluorescence, the fluorescence yield from PS1 does not vary with light intensity nor temperature [23]. Correspondingly, little is known about whether and how the function of PS1 might change with temperature, or the role of this photosystem in coral bleaching. Measuring equipment has recently been developed to monitor PS1 activity through changes in light absorbance in the near-infrared wavelengths (830nm) associated with changes in the oxidation state of PS1 [23].

This study used a multi-faceted approach to quantify changes in photobiology of the symbionts from two common coral species, *Turbinaria reniformis* and *Stylophora pistillata* (hereafter *Stylophora* and *Turbinaria*), in response to thermal stress. We quantified how exposure to thermal stress affected net coral colony photosynthesis and respiration using oxygen measurements, and measured the underlying activities of both PS2 and PS1 using chlorophyll fluorescence and light absorption techniques. We analysed the effects of thermal stress on zooxanthellae density and chlorophyll content, and used immunoblotting techniques to quantify the content of representative proteins from PS2, PS1 and Rubisco complexes within the zooxanthellae. The severity of bleaching is exacerbated by the degree to which light intensity exceeds the light levels to which coral symbionts are acclimatized [24], and is also sensitive to nutrient limitation at high light levels [25]. Despite the presence of *Symbiodinium* within their tissue, coral polyps retain their capacity to capture prey to support their nutrient and metabolic requirements. For certain coral species increased rates of heterotrophic feeding can compensate for reduced photosynthesis during and after bleaching events [26], [27], [28]. Therefore, we conducted our thermal stress trials under moderate and high light, and in the presence and absence of prey for the coral hosts. We sought to determine how both symbiont photosystems respond to the light, temperature and feeding regimes imposed upon their hosts, and in particular whether feeding modulates the responses of corals to temperature stress.

## Methods

### Experimental setting

Three separate experiments were conducted with the same overall design but differing in the light and feeding regime under which corals were incubated. These experiments were designed and implemented to reflect benign (low light, adequate food) compared with increasingly stressful conditions (food deprivation, food deprivation plus high-light stress). Coral colonies were sourced from the Red Sea and maintained in culture at the Centre Scientifique de Monaco. From these stocks, 3 sets of 24 small experimental colonies (nubbins) per species were created several weeks prior to each experiment, and allowed to heal under the culture conditions. Subsequently, colonies were moved into 4 glass aquaria (18 l volume) with 2 tanks for each temperature treatment, and were maintained under the conditions specific to each experiment for a period of two weeks. Light (120 µmol photons m^−2^ s^−1^ in Experiments 1 and 2; 250 µmol photons m^−2^ s^−1^ in Experiment 3) was set to the required level using metal halide lamps and neutral-density shade screens, and measured using a LI-COR data logger (LI-1000) with a spherical quantum sensor. The ‘high-light’ treatment corresponds to ≈ 11 mol photons m^−2^ d^−1^ and is ecologically relevant both at the depths at which the study species occur in the field [29], and the light levels under which they are grown in our laboratory. Colonies in the ‘fed’ treatments (Experiment 1) were provided with *Artemia salina* nauplii twice weekly, directly into the aquaria at a feeding density of approximately 1000 prey l^−1^. Following the acclimation period in each experiment, two tanks were maintained at 26°C while temperature in the other tanks was increased to 32°C and maintained at that level for 10 – 13 days (Table 1). Temperature was monitored daily and was maintained at 26°C or 32°C ± 1.5°C using thermostat-regulated aquarium heaters (Visy-Therm, 300W). Each experiment was terminated when paling of tissue (relative to the source stock of coral colonies maintained under long-term culture conditions in our laboratory) was visually observed in 50% of the nubbins from either species. Paling was mainly observed in *Turbinaria*, with nubbins of *Stylophora* only changing slightly in color over the experimental period. We adopted this conservative approach to implementing the heat-stress in order to ensure that adequate symbiont densities for assays of symbiont photophysiology and photosynthetic protein subunit content were present in all samples. On the day that each experiment was terminated, fluorometry assays were performed on 6 nubbins from the 32°C treatment (3 from each tank) after which the colonies were immediately frozen at −80°C for subsequent protein assays. At the same time, photosynthesis-irradiance curves were measured for the remaining colonies from this treatment after which colonies were frozen at −20°C for subsequent measurements of symbiont density and chlorophyll content. The same sets of measurements were made on colonies from the control temperature on the following day.

**Table 1:** Analysis of variance testing the effects of temperature and experimental conditions on chlorophyll content, photosynthesis and respiration of *Turbinaria reniformis* and *Stylophora pistillata*.

Parameter	Species	Factor	DF	F	p
Chlorophyll (mg cm^−2^)	*Turbinaria*	Temperature	1,30	5.5	< 0.05
		Experiment	2,30	13	< 0.001
		Temp x Experiment	2,30	13.9	< 0.05
	*Stylophora*	Temperature	1,30	0.11	0.92
		Experiment	2,30	11	<0.001
		Temp x Experiment	2,30	1.2	0.29
Photosynthesis ( µmol O_2_ cm^−2 ^h^−1^)	*Turbinaria*	Temperature	1,30	19	< 0.05
		Experiment	2,30	14	< 0.001
		Temp x Experiment	2,30	3.1	0.06
	*Stylophora*	Temperature	1,30	67	<0.001
		Experiment	2,30	67	<0.001
		Temp x Experiment	2,30	16	<0.001
Respiration ( µmol O_2_ cm^−2 ^h^−1^)	*Turbinaria*	Temperature	1,30	2.2	0.15
		Experiment	2,30	10.3	< 0.001
		Temp x Experiment	2,30	2.1	0.14
	*Stylophora*	Temperature	1,30	1.0	0.32
		Experiment	2,30	8.2	<0.01
		Temp x Experiment	2,30	2.3	0.12

DF denotes degrees of freedom for each effect. Temperature refers to the experimental temperature treatment (two levels: 26°C and 32°C) and experiment refers to the different light and feeding treatments (three levels: low light fed, low light unfed, high light unfed).

### Colony photosynthesis and respiration

Rates of photosynthesis and respiration were measured using a set of three temperature-controlled respirometry chambers (50 ml volume) coupled with a Strathkelvin oxygen electrode system (Strathkelvin 928 meter with computer interface). Electrodes were calibrated at the incubation temperature immediately prior to respirometry measurements using N_2_- and air-bubbled seawater as 0% and 100% oxygen saturation values respectively. Temperature was maintained at 26°C or 32°C during all incubations and the chambers were stirred using magnetic stirrers. A metal halide lamp mounted on a sliding platform was used as a light source. For each of 6 colonies per treatment, respiration (oxygen consumption) was measured during incubation in darkness (30 mins). Subsequently, light intensity was increased to 125 µmol photons m^−2^ s^−1^ then 450 µmol photons m^−2^ s^−1^, and photosynthesis rates were measured at each light level (15 min incubation).

### Chlorophyll and symbiont quantifications

To estimate zooxanthella density and chlorophyll concentration, tissue was removed from the skeleton of each of 6 colonies per treatment, using an air-pick, and collected in a beaker with 7 ml of 0.45 µm filtered seawater. The tissue slurry was homogenized using a Potter tissue grinder and a 1 ml sub-sample was taken for zooxanthellae counts. Symbiont densities in samples of volume 100–300 µl were counted using an inverted microscope (Leica, Wetzlar, Germany) and the Histolab 5.2.3 image analysis software (Microvision, Every, France). The remaining tissue slurry for each colony was centrifuged at 8000 g for 10 min. Subsequently, the supernatant was removed and the zooxanthellae re-suspended in 5 ml of acetone for extraction of chlorophylls *a* and c2. Samples were kept in darkness at 4°C for a period of 24 h to ensure total extraction. Chlorophyll content was determined using a spectrophotometry method. Samples were centrifuged for 15 min at 11000 g before absorbance was measured at 750, 663 and 630 nm. Absorbance at 750 nm was used to control for sample turbidity, and chlorophyll content was calculated from A663 and A630 [30]. Patterns of among-treatment variation in symbiont density were qualitatively similar to, and positively correlated with, the observed variation in chlorophyll concentration and, hence, symbiont density data are not presented here.

### Fluorometry – PS2 fluorescence and PS1 absorbance

**Figure 1:**
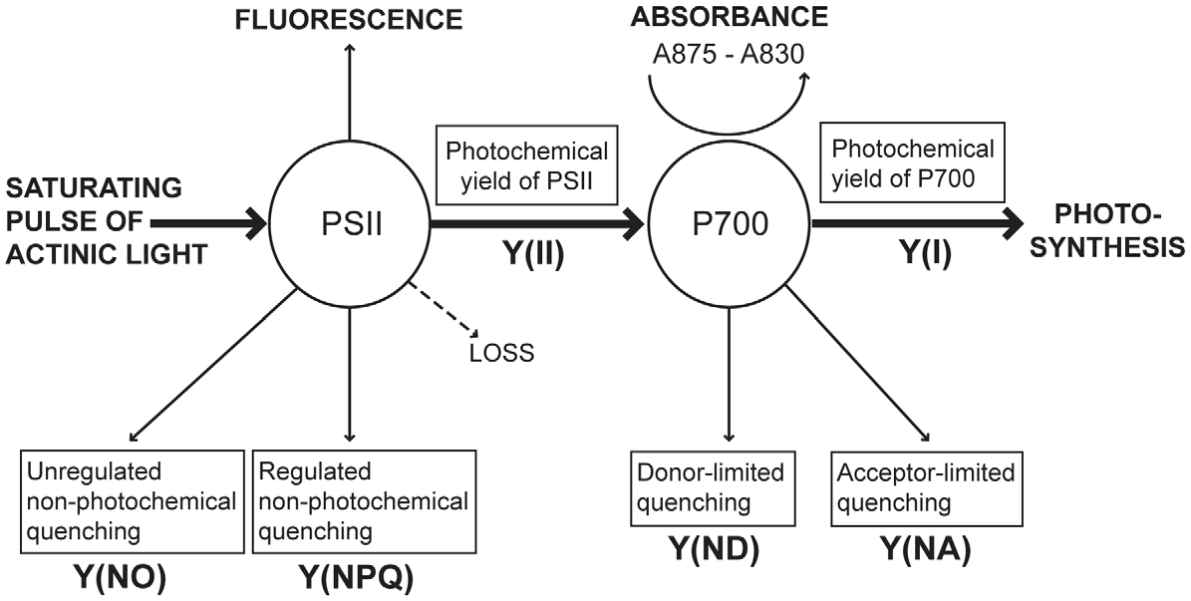
Photochemistry parameters measured using the DualPAM. Photosystem 2 (PS2) parameters are measured from chlorophyll fluorescence and PS1 parameters are based on absorbance at 875 compared with 830 nm. Abbreviated variable names correspond to definitions in boxes and are referred to throughout the paper. Details of measurement are outlined in Methods section “Fluorometry – PS2 fluorescence and PS1 absorbance”.

*In vivo*, chlorophyll takes the form of pigment/protein complexes that are embedded in thylakoid membranes and funnel excitation energy to photosynthesis reaction centers (RCs). Recent advances in fluorometry technology lead to the development of the Dual-PAM (www.walz.com) that allows simultaneous measurement of both PS2 chlorophyll fluorescence and PS1 light absorbance. In darkness, when all RCs are open, chlorophyll fluorescence from PS2 is minimal (denoted F_o_). Conversely, when all RCs are closed after illumination with a saturating pulse, chlorophyll fluorescence is maximal (denoted F_m_) and the ratio of F_m_ to F_o_ indicates the maximal quantum yield of PS2. Under illumination with photosynthetically active radiation, when a subsequent saturating pulse is applied, the maximal and minimal fluorescence yields (F_m_′ and F_o_′) change due to the induction of non-photosynthetic quenching mechanisms [17]. The saturation pulse method allows the partitioning of absorbed excitation energy between light used for photochemistry, Y(II), and excitation that is dissipated (Fig. 1). The latter component is split into regulated and non-regulated processes. Regulated dissipation, Y(NPQ) is thought to function to protect the photosynthetic units from damage due to excess light absorption. Non-regulated dissipation, Y(NO), is a consequence of PS2 reaction center closures due to inhibition of electron transport. A high value of Y(NPQ) indicates that irradiance is excessive but shows that the sample has retained the regulatory mechanisms to protect its photosynthetic apparatus. A high value of Y(NO) indicates that both the photochemical energy conversion and the protective regulatory mechanisms are insufficient to process excitation energy.

The activity of PS1 is quantified through oxidation-dependent changes in light absorbance at 830nm, in comparison to a reference absorbance measure at 875 nm [23], which occurs upon illumination. In analogy to F_m_, P_m_ represents the maximal change of the PS1 signal upon transformation of PS1 from a fully reduced to fully oxidized state, and is measured by application of Far-Red pre-illumination that activates PS1 and draws electrons from the inter-system electron transport chain, followed by a saturating pulse that drives PS1 to full oxidation. Similarly to PS2 F_m_′, maximal PS1 change under illumination, P_m_′, represents the maximal change of the PS1 signal after a saturating pulse. P_m_′ is lower than P_m_, reflecting acceptor-side limitation of PS1 [23].

The fraction of overall PS1 that is reduced at a given time is represented by the parameter PS1 red, and can vary between 0 (PS1 fully oxidised, signal at P_m_′ or P_m_) and 1 (PS1 fully reduced, normal dark state). Even under illuminated conditions, PS1 can be transiently driven to full reduction by electrons arriving from PS2 after application of a pulse of white light to saturate PS2 photochemistry. Only those PS1 centres with reduced PS1 can contribute to the instantaneous photochemical quantum yield of PS1, referred to as Y(I). However, not all of the reduced centres do in fact contribute to Y(I) because not only the donor side (PS2) but also the acceptor side (photochemistry) can be limiting [23]. The photochemical quantum yield of PS1, Y(I) is defined by the fraction of overall PS1 that is reduced and not acceptor-limited. It is calculated from the fraction of overall PS1 that is oxidised, Y(ND), and the fraction of overall PS1 that cannot be oxidised due to a lack of acceptors, Y(NA):
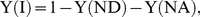
(1)Y(ND) is a measure of donor-side limitation and reflects down-regulation or damage to PS2 and is calculated from PS1 red. as:
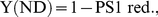
(2)Y(NA) also measures non-photochemical yield of PS1 but represents the fraction of PS1 that cannot be oxidised during a saturating pulse because of the lack of acceptors. Y(NA) is calculated analogously to NPQ because acceptor-limited PS1 centres transfer excitation energy into heat:
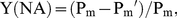
(3)In this study, PS2 and PS1 parameters were quantified for each experimental colony. To do this, colonies were transferred into seawater in a small glass container and placed inside a dark box to acclimate for 15 minutes prior to measurement. Dark-adapted PS2 rapid-light curves (RLC) were conducted for each colony. Colonies were then allowed to ‘rest’ for 10 minutes before PS1 RLC were measured. These two RLC were conducted in darkness, and PS1 measurements were taken from a different region of the colony than PS2 measurements to avoid potential effects of prior exposure to saturating light pulses on PS1 photochemistry.

### Quantification of PS2, PS1 and Rubisco

Contents of photosystem proteins were determined using quantitative immunoblotting [31], [32]. The target proteins were PsbA, PsaC and RbcL for the quantification of PS2, PS1 and Rubisco content respectively. Six coral samples per treatment were thawed and tissue was separated from the skeleton using the Water-Pik technique. Zooxanthellae were separated from the host tissue by two centrifugations (10 min at 8000g) in 2 mL of 0.2 µm filtered seawater. For each centrifugation, supernatant was withdrawn and the zooxanthellae pellets flash-frozen in liquid nitrogen. Following purification, samples were kept at −80°C until analysed.

To extract total protein from the samples, frozen zooxanthellae pellets were re-suspended in 500 µl of a denaturating extraction buffer, and underwent two rounds of sonication followed by flash freezing in liquid nitrogen as previously described [31], [32]. The total protein concentration was quantified using a modified Lowry assay (Bio-Rad DC) with bovine gamma globulin as a comparative protein standard. As described previously [32], samples were prepared, run on 4–12% gradient Bis-Tris NuPAGE gels (Invitrogen) in MES-SDS running buffer (Invitrogen) at 200V for 40 min, and transferred to polyvinylidene difluoride (PVDF) membranes. Membranes were immunoblotted as previously described [28] using specific primary antibodies for PsbA (AgriSera www.agrisera.se, AS01 016), PsaC (AS10 939) and RbcL (AS03 037) and horseradish peroxidise-conjugated secondary antibody solutions. Blots were developed with ECL Advance detection reagent (GE Healthcare) and images were obtained using a CCD imager (FluorSMax, Rad) and Quantity One software (Bio-Rad). Protein levels in immunoblots were estimated using standard curves created using protein standards run in parallel with sample protein extracts; PsbA (AgriSera AS01 016S), PsaC (AS04 042S), and RbcL (AS01 017S).

### Data analysis

Analysis of variance (ANOVA) was used to quantify the effects of experimental treatment and temperature on chlorophyll *a* concentration, photosynthesis, respiration and contents of PsbA, PsaC and RbcL. In these analyses, ‘experiment’ and ‘temperature’ were treated as fixed factors and analyses therefore assess whether the experimental conditions affected the responses of coral colonies, and whether any temperature effects were consistent between experiments. Analyses were conducted separately by species and data were log-transformed where required to meet normality and homogeneity of variances. Chlorophyll *a*, photosynthesis and respiration data were normalised to colony surface area for these analyses.

Fluorometry assays of photosynthesis generate large datasets with many correlated variables that explain different, yet related, components of photophysiology. We did not know *a priori* how strongly PS1 function would vary with temperature nor whether including PS1 measurements contributed additional information regarding changes in photochemistry under combinations of temperature stress and experimental conditions. Therefore, we used principal components analysis (PCA) to extract the main components of variation in photophysiology, and analysed whether and how the biological factors (species identity, incubation conditions and temperature) related to these components. This approach was used to visualise the strongest of the observed effects on the overall symbiont photophysiological response rather than attempting to analyse the effects of each factor on each variables because the latter analyses can be confounded by co-variation among multiple photophysiological variables. To extract as much information as possible from the PS1 and PS2 rapid-light curves measured for each colony, we fitted empirical equations to the data describing the relationship between light intensity (during RLC) and each variable described in “*Fluorometry – PS2 fluorescence and PS1 absorbance*”. The PCA included 16 variables, as follows: maximum Y(II), maximum rETR(II), initial rate of increase of rETR(II), photoinhibition of rETR(II), minimum Y(NO), maximum Y(NO), rate of change of Y(NO), maximum Y(NPQ), rate of change of Y(NPQ), maximum Y(I), rate of change of Y(I), maximum rETR(I), initial rate of increase of rETR(I), maximum Y(ND), minimum Y(ND), rate of change of Y(ND) and maximum Y(NA).

## Results

### Pigment and protein content

**Figure 2:**
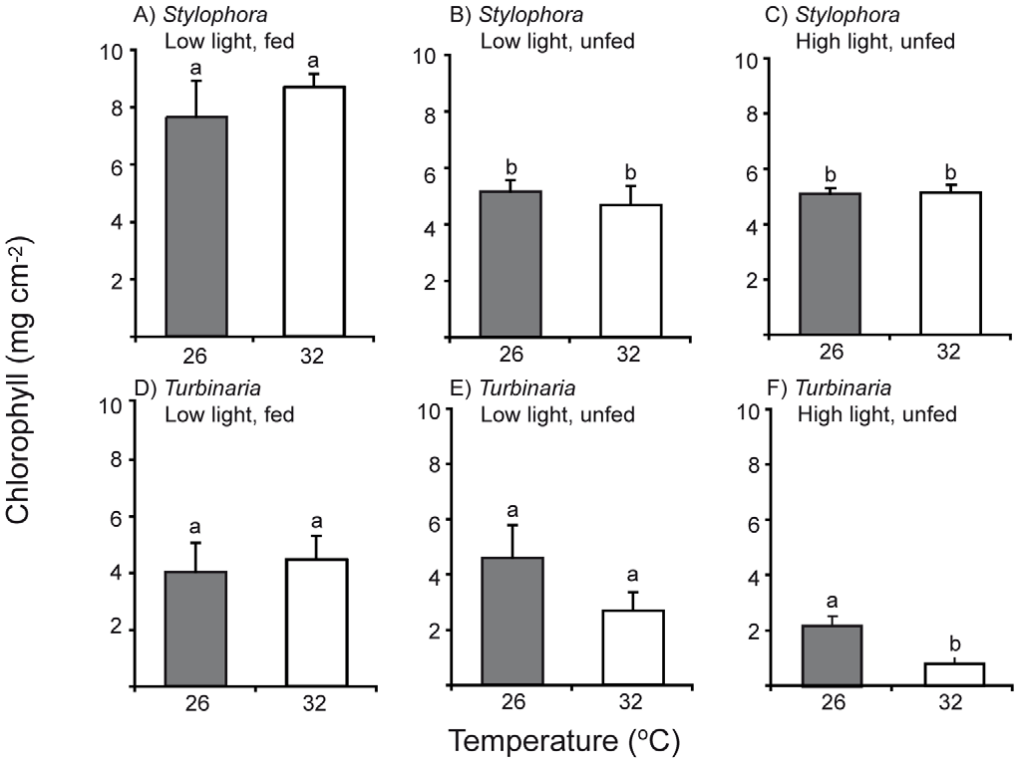
Chlorophyll levels for colonies of *Turbinaria reniformis* and *Stylophora pistillata* incubated at control (26°C) and high (32°C) temperatures under different light levels and feeding regimes. Lower case letters denote statistically homogeneous subsets across temperature and experiment but within species, as shown in Table 1, and error bars represent standard error.

For *Stylophora*, chlorophyll *a* (chl) content was mainly affected by food availability: chl was significantly higher in fed nubbins of this species (Fig. 2A, Table 1) than in unfed nubbins maintained at the same (Fig. 2B) or higher light levels (Fig. 2C). Temperature stress caused by incubation at 32°C, even for a period of 10 – 14 days, did not cause a decrease in chl content for these colonies (Table 1). Fed colonies of *Turbinaria* also tended to have higher chl content than unfed colonies, and chl content for this species tended to be lower under the high light incubation, although these trends were not statistically significant( (Fig. 2D, Table 1). The effect of temperature on chl content of *Turbinaria* colonies varied across the incubation conditions (Table 1). Chl content was significantly lower at 32°C for unfed colonies maintained under high light (Fig. 2F). Overall, chlorophyll content of *Stylophora* was enhanced by feeding but was largely independent of light level and temperature stress.

**Figure 3:**
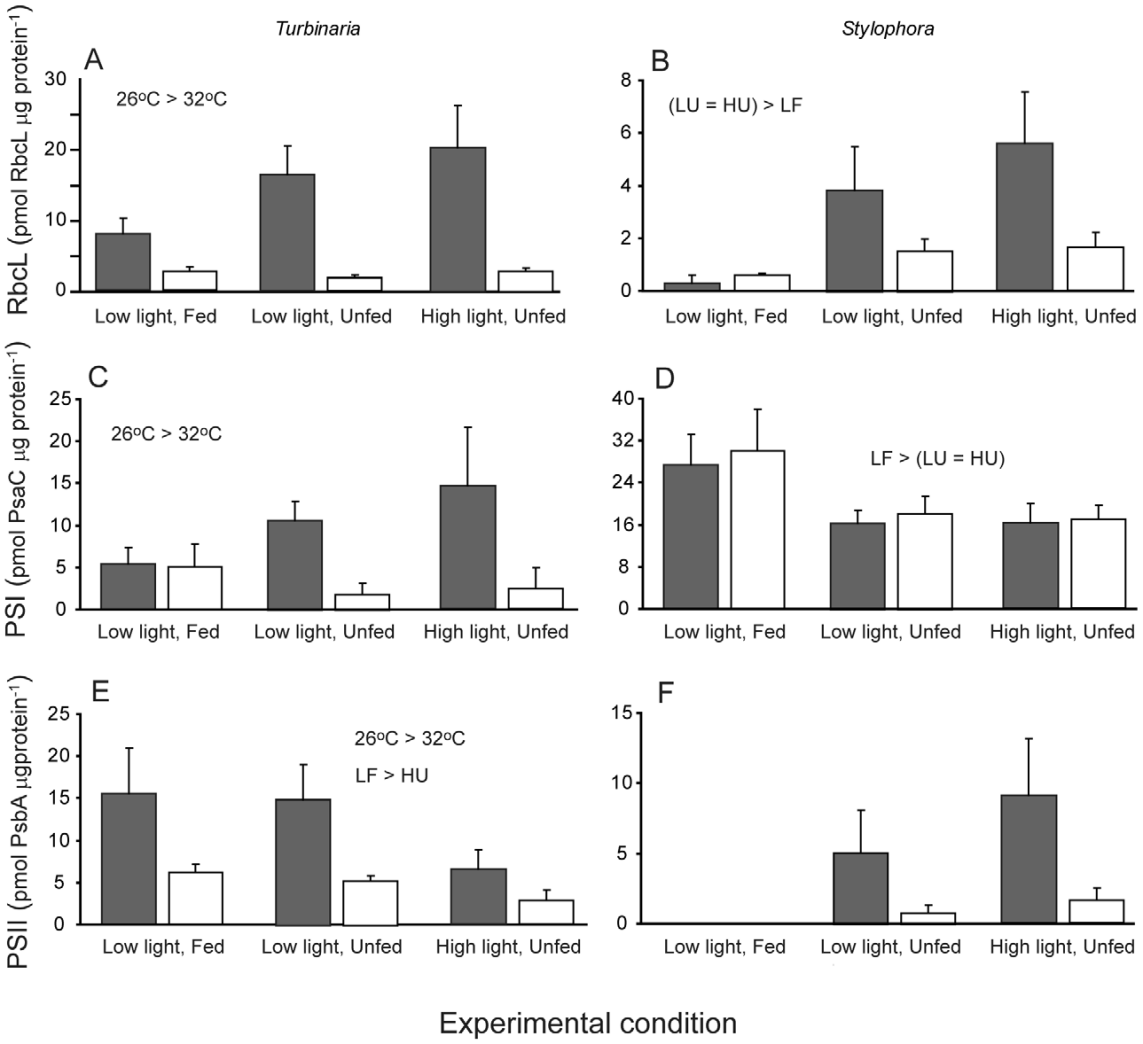
Protein contents for colonies of *Turbinaria reniformis* and *Stylophora pistillata* incubated at control (26°C, grey bars) and high (32°C, open bars) temperatures under different light levels and feeding regimes. Summary statistics are provided in Table 2 and error bars represent standard error.

We used quantitative immunoblotting against PsbA (PS2), PsaC (PS1) and RbcL (Rubisco) proteins to investigate changes in components of the photosynthetic machinery in response to experimental conditions and temperature stress (Fig 3, Table 2). In *Stylophora* levels of the photosynthetic complexes were not strongly affected by temperature and instead varied mainly according to the light and feeding regimes (Table 2). For *Stylophora*, RbcL levels were higher but PsaC levels were lower in unfed compared with fed corals (Fig 3B and D, Table 2). Two-way ANOVA could not be reliably used to assess treatment effects on PsbA content in *Stylophora* because levels of this protein were so low as to be undetectable under low light, fed conditions, leading to strong heterogeneity of variances that could not be corrected by data transformation. However, in unfed incubations, PsbA levels were much lower at 32°C than at 26°C (Fig. 3F), although this trend was not statistically significant due to high-variability within-groups (Table 2). In *Turbinaria*, contents of all these proteins were significantly lower at high temperature (Table 2, Fig. 3A, C, E). For *Turbinaria* PsbA levels were higher in fed corals maintained under low light compared to unfed corals maintained under high light (Fig. 3C). Modulation of the RbcL:PsbA ratio, or PsaC:PsbA ratio, generally indicates adaptation of the photosynthetic apparatus to ambient light intensity with these ratios tending to increase under high growth irradiances [32]. In unfed nubbins of *Stylophora* at 26°C neither ratio changed with growth irradiances (RbcL:PsbA ratio of 3.3 and 3.2 and PsaC:PsbA ratio of 6.9 and 7.2 in low and high light respectively). Conversely, in *Turbinaria* nubbins, both ratios increased with growth irradiances: from 0.9 in low light to 2.8 in high light for RbcL:PsbA and from 0.9 to 3.7 for PsaC:PsbA.

**Table 2:** Analysis of variance testing the effects of temperature and experimental conditions on three photosystem proteins Rubisco (RbcL), PS2 (PsbA) and PS1 (PsaC).

Parameter	Species	Factor	DF	F	p
RbcL (nmol µg^−1^)	*Turbinaria* (a)	Temperature	1,30	51	< 0.001
		Experiment	2,30	1.8	0.18
	*Stylophora* (a)	Temperature	1,32	2.6	0.11
		Experiment	2,32	7.5	<0.01
PsaC (PS1) (nmol µg^−1^)	*Turbinaria*	Temperature	1,32	6.1	< 0.05
		Experiment	2,32	0.6	0.57
	*Stylophora*	Temperature	1,32	0.2	0.66
		Experiment	2,32	4.4	<0.05
PsbA (PS2) (nmol µg^−1^)	*Turbinaria* (b)	Temperature	1,30	6.5	<0.05
		Experiment	2,30	4.5	<0.05
	*Stylophora* (c)	Temperature	1,21	3.8	0.07
		Experiment	2,21	0.8	0.39

In analysis marked (a) data were log transformed to homogenise variances, in (b) data were rank-transformed and in (c) only experiments 2 and 3 were compared. Temperature refers to the experimental temperature treatment (two levels: 26°C and 32°C) and experiment refers to the different light and feeding treatments (three levels: low light fed, low light unfed, high light unfed).

### Activity of photosystem II and photosystem I

**Figure 4:**
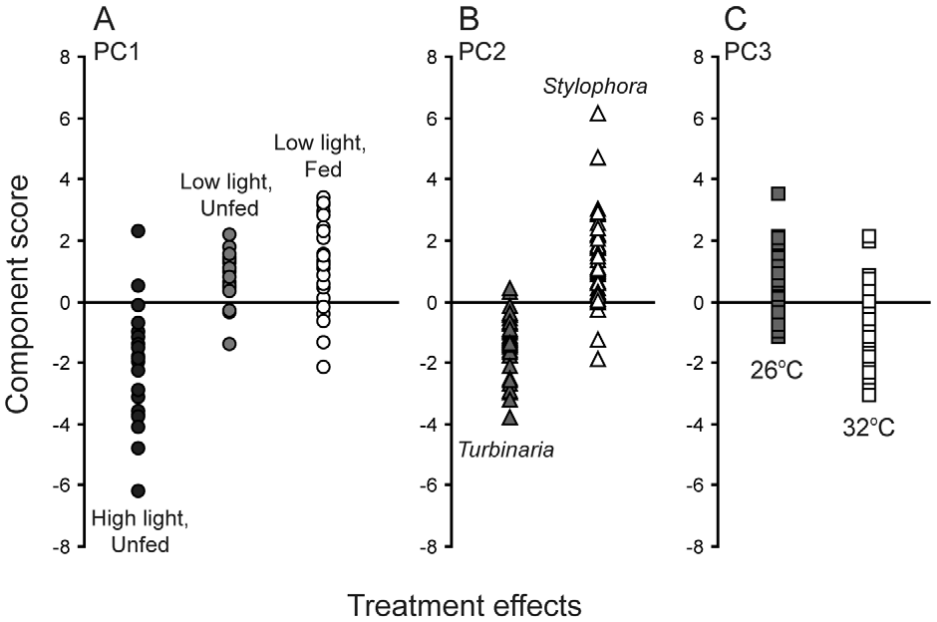
Component scores from Principal Components Analysis of photophysiology data. Points show scores for individual coral colonies and indicate experiment, species and temperature dependence of scores on the first three principal components respectively (PC1, PC2 and PC3). Summary statistics are provided in Table 3.

Principal Components Analysis (PCA) was used to determine which of the measured fluorescence and absorbance parameters best captured variation in PS2 and PS1 photophysiology under the set of experimental conditions. Three main principal components were identified (Fig. 4, Table 3) that explained more than 52% of the variance in overall photosystem activity. The first principal component (PC1) differentiated between the three experiments (Fig. 4, PC1) primarily on the basis of light intensity: Experiment 3 (high light, 250 µmoles photons m^−2^ s^−1^) presented negative component scores in comparison to the other two experiments (low light, 120 µmoles photons m^−2^ s^−1^). Principal component 2 (PC2) was governed by species identify (Fig. 4, PC2) and showed that photochemistry of *Turbinaria* (negative scores) was differently affected by feeding, light intensity and temperature compared to that of *Stylophora* (positive scores). Finally, principal component 3 (PC3) separated coral nubbins on the basis of incubation temperature, with corals incubated at 26°C presenting more positive scores on this axis compared to those kept at 32°C (Fig 4, PC3).

**Table 3:** Variable loadings and correlations between variables and components (PC1 – 3) defined through Principal Components Analysis of photochemical parameters.

Component	Photochemistry variable	Loading	Correlation	t	p
**PC1: Light**	Maximum Y(II)	0.39	0.78	10.3	< 0.001
	Minimum Y(NO)	−0.39	−0.77	−10.0	< 0.001
	Maximum Y(NO)	0.34	0.67	7.6	< 0.001
	Maximum Y(NPQ)	−0.30	−0.60	−6.3	< 0.001
	Y(NPQ) rate of change	−0.33	−0.65	−7.2	< 0.001
**PC2: Species**	Maximum rETR(II)	0.47	0.82	12.2	< 0.001
	rETR(II) rate of change	−0.41	−0.71	−8.5	< 0.001
	Maximum Y(ND)	0.35	0.61	6.5	< 0.001
	Minimum Y(ND)	0.31	0.54	5.4	< 0.001
**PC3: Temperature**	Maximum Y(NPQ)	0.30	0.40	3.6	< 0.01
	Maximum rETR(I)	−0.49	−0.66	−7.4	< 0.001
	rETR(I) rate of change	−0.57	−0.77	10.0	< 0.001
	Minimum Y(ND)	−0.38	−0.51	−5.0	< 0.001

Photochemistry variables and abbreviations are as described in Figure 1. Principal component variances and standard deviations were: 23% and 1.99 respectively for PC1, 18% and 1.76 respectively for PC2, and 11% and 1.35 respectively for PC3.

**Figure 5:**
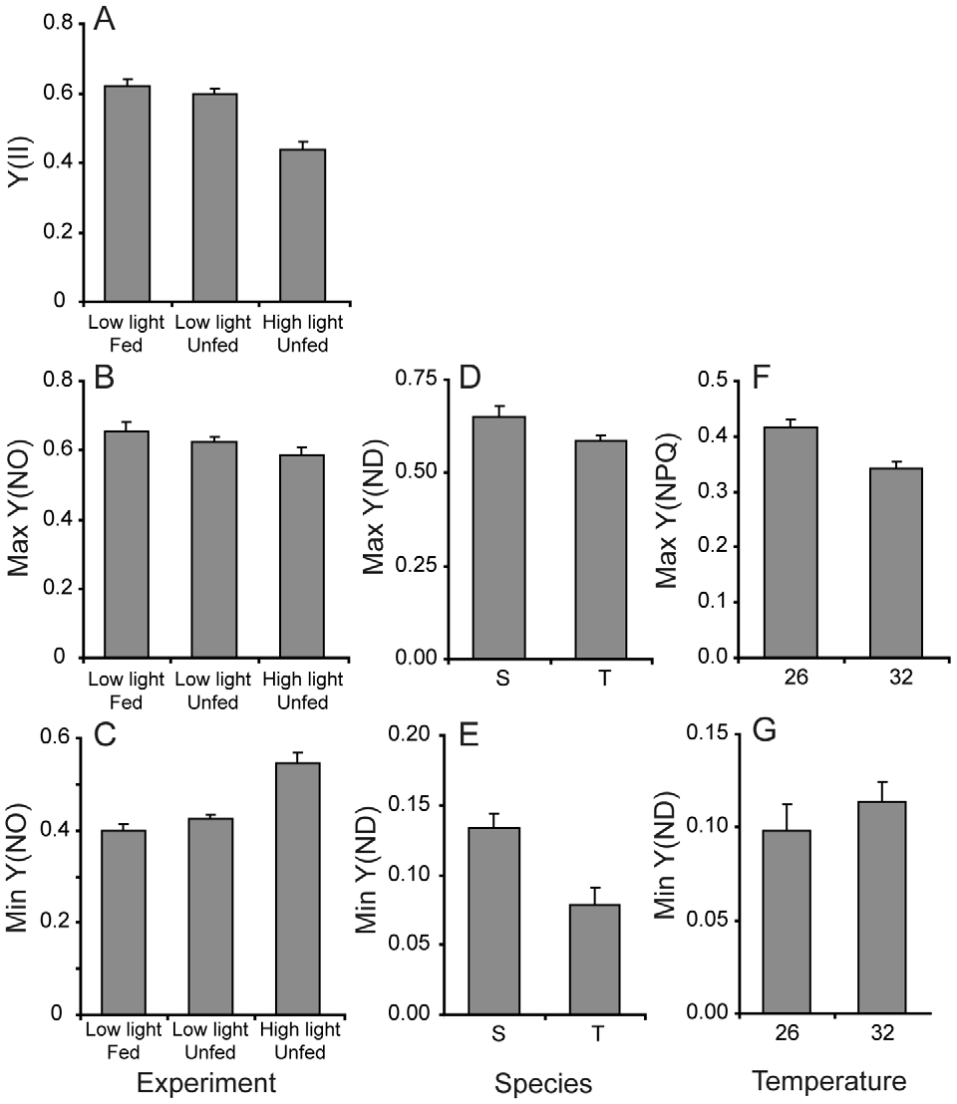
Variation in photophysiology parameters identified through principal components analysis as having a strong effect on overall photochemistry. Data for each variable are pooled and plotted according to the effect identified for the principal component with which they were strongly associated (Fig 4, Table 3). For example, Y(ND) was strongly related to PC2 which differentiated between the study species *Turbinaria reniformis* (T) and *Stylophora pistillata* (S). 26 and 32 denote the two different incubation temperatures of 26°C and 32°C. Bars show means and standard errors.

**Figure 6:**
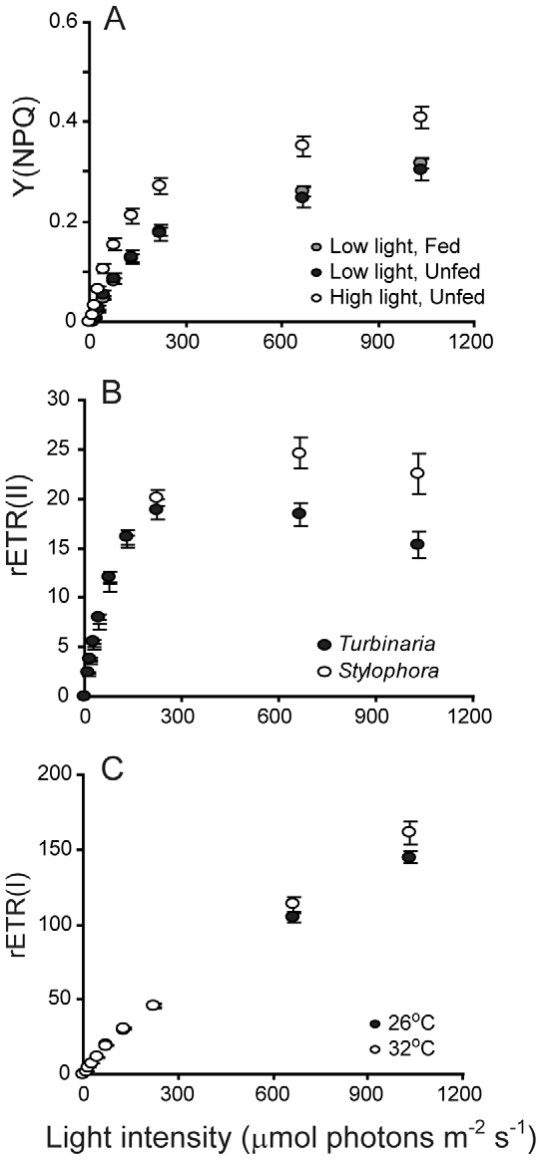
Rapid light curves showing variation in photophysiology parameters identified through principal components analysis as having a strong effect on overall photochemistry. Data for each variable are pooled and plotted according to the effect identified for the principal component with which they were strongly associated (Table 3). In A), data are pooled across species and temperatures because Y(NPQ) was strongly related to PC1 which differentiated between experiments. In B) data are pooled across temperatures and experiments and in C) data are pooled across species and experiments. Points show means and error bars depict standard error.

Particular aspects of photochemistry changed in response to different experimental conditions and temperature stress (Table 3, Fig 5 and 6). PC1, which mostly represents the light effect, captured the largest proportion (23%) of the overall variance in PS2 parameters. PC1 was significantly correlated with several measures of PS2 activity, including the maximum PS2 quantum yield, Y(II), and the unregulated, Y(NO), and regulated non-photochemical quenching, Y(NPQ). Corals maintained under high light had the lowest Y(II) (Fig 5A) and the highest levels of unregulated quenching (minimum Y(NO), Fig 5C). Nevertheless, Y(NO) did not increase during exposure to increasing measurement light intensity for these corals (Fig 5B). In other words, high-light corals had higher ‘background’ levels of unregulated quenching, but increasing measuring light levels did not cause the same immediate increase in Y(NO) as was observed for the low-light corals. High-light corals also had higher levels of Y(NPQ) compared to corals maintained under low light (Fig 6A). Both parameters indicate that the incubation light intensity was excessive for the high-light corals and induced an up-regulation of photo-protective mechanisms, indicated by Y(NPQ). However, this photoprotection was not activated in corals kept at low light (Fig 5B) and in this case was not sufficient to prevent damage during exposure to high irradiance shown by high values of maximum Y(NO), (Fig. 5B). PS1 activity was not strongly associated with PC1.

The second component, PC2, represented between-species variation in photochemistry in response to feeding, light and temperature, and explained 19% of the variance (Table 3). Changes in the activity of both PS2 and PS1 differentiated the two study species (Table 3). Specifically, *Stylophora* tended to have lower maximum quantum yield but generated higher PS2 electron transport rates at high light intensities (rETR(II)) with little evidence of photoinhibition at very high irradiances (Fig 6B). On the other hand, *Turbinaria* presented a higher maximal quantum yield (0.62±s.e. 0.02 compared with 0.49±s.e. 0.02 for *Stylophora*) but generally had lower rETR(II) across a broad range of light intensity and showed evidence of photoinhibition at very high light levels (Fig. 6B). Consistent with the maximum quantum yield data, *Turbinaria* showed lower PS1 donor limitation at low light (Table 3, Fig 5E) compared with *Stylophora*. However, Y(ND) was also lower for *Turbinaria* compared with *Stylophora* when measured under high-light intensity (Fig 5D), contrary to our finding of higher PS2 photoinhibition based on PS2 electron transport rates for this species (Fig 6B).

The third principal component, PC3, primarily captured the effect of temperature on PS1 and PS2 photochemistry. Conversely to PC1 and PC2, which were mostly associated with changes in PS2 parameters, PC3 was most strongly correlated with PS1 activity (Table 3). Contrary to our expectation, the maximum electron transport rate through PS1, rETR(I), was higher in corals exposed to 32°C than at 26°C (Fig 6C) but increased more rapidly towards this maximum for corals grown under the lower temperature (negative correlation between rate of change of rETR(I) and PC3, Table 3). The latter result supports our finding that baseline donor-side limitation of PS1 activity, Y(ND) minimum, was higher for the corals grown at high temperature (Table 3, Fig 5G). That is, the lower donor limitation at low light observed in the control corals translated to higher electron transport through PS1 at low light intensities during rapid light curves. Finally, the capacity for non-photochemical quenching at PS2, Y(NPQ) was smaller for corals kept at 32°C compared with 26°C (Fig 5F).

**Figure 7:**
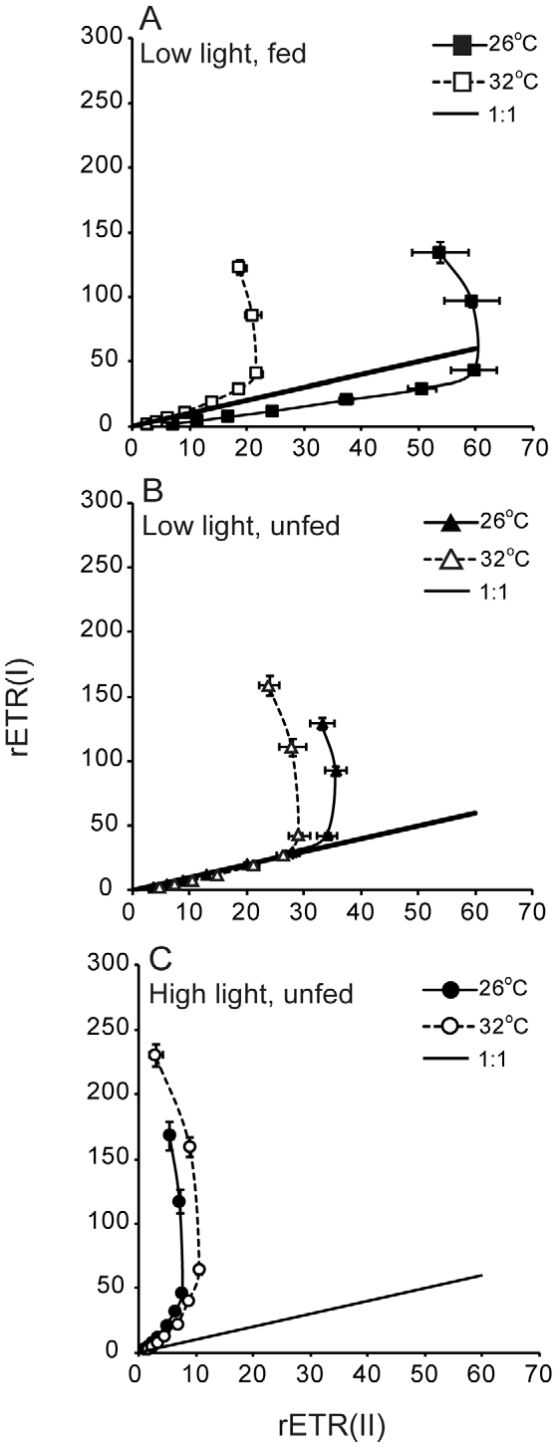
Electron transport through PS2 versus that through PS1, corrected for relative contents of each photosystem based on protein quantifications. A, B and C are the results obtained with *Turbinaria reniformis* under A) low light, fed; B) low light unfed; c) high light unfed conditions. Error bars represent standard error.

Electron transport through PS1 and through PS2, corrected for the relative quantity of PS1 versus PS2 reaction centres based on protein quantifications, showed near-linear correlations under moderate light, consistent with linear electron transport through PS2 and PS1. For unfed *Turbinaria* growing under low light rETR(I) indeed showed a near 1∶1 correlation with rETR(II), as expected for linear photosynthetic electron transport (Fig 7A, B). Under all growth conditions and at high measuring light intensity rETR(I) continued to increase while rETR(II) eventually saturated, indicative of increased cyclic electron transport through PS1, decoupled from linear electron transport through PS2 and PS1. *Turbinaria* grown under low light maintained linear electron transport over a wider range of light at 26°C than at 32°C, consistent with inhibition of PS2 at 32°C. For *Turbinaria* growing under high light rETR(II) was inhibited while rETR(I) was not, leading to a departure from the 1∶1 correlation expected for linear photosynthetic electron transport. (Fig 7C). Overall, provision of food enhanced rETR(II) relative to rETR(I) but growth under high light intensity inhibited rETR(II).

### Colony photosynthesis and respiration

**Figure 8:**
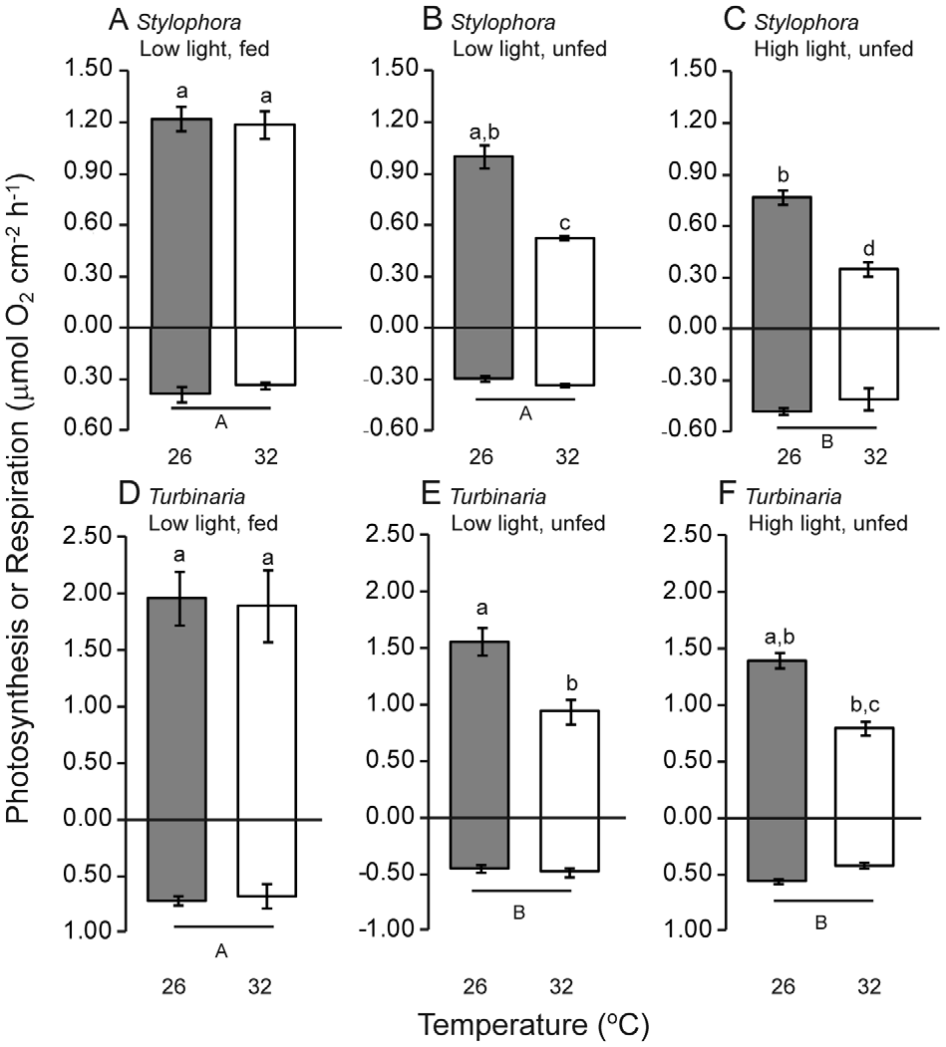
Photosynthesis and respiration for colonies of *Turbinaria reniformis* and *Stylophora pistillata* incubated at control (26°C) and high (32°C) temperatures under different light levels and feeding regimes. Lower case letters denote statistically homogeneous subsets across temperature and experiment but within species, as shown in Table 1. Positive values show rate of photosynthesis at 450 µmol photons m^−2^ s^−1^and negative values show rate of respiration in darkness. Error bars represent standard error.

**Figure 9:**
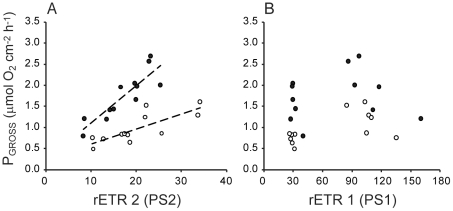
Relationship between gross photosynthesis and relative electron transport. In A) relative electron transport through PS2 is shown, and in B) relative electron transport through PS1. Points represent individual colonies, filled circles show values for *Turbinaria reniformis* and open circles show values for *Stylophora pistillata*. Lines in A) are trend lines representing significant correlations.

For unfed colonies of both *Stylophora* and *Turbinaria* rates of photosynthesis were lower at 32°C compared to 26°C (Table 1, Fig 8). Growth under high light also resulted in slightly lower rates of net photosynthesis (Fig 8). Under low light the fed colonies of both *Stylophora* and *Turbinaria* maintained photosynthesis in the face of the 32°C temperature stress (Fig 8A and D). For both *Stylophora* and *Turbinaria* experimental conditions significantly influenced respiration rates. For *Stylophora* colony respiration increased for colonies grown under high-light intensity (Fig 8A–C) whereas colony respiration in *Turbinaria* was significantly lower for unfed compared to fed colonies at both growth light levels (Fig. 8D–F). At the light intensity within the rapid light curve that was equivalent to the light intensity during oxygen respirometry measurements electron transport through PS2 showed a positive linear correlation with gross colony photosynthesis for *Stylophora* and *Turbinaria* (Fig 9A). Pearson’s correlation coefficients for these analyses were equal to 0.79 for *Stylophora* (t_10_ = 4.1, p<0.01) and 0.87 for *Turbinaria* (t_10_ = 5.7, p<0.001). Conversely, electron flux through PS1 showed no relation with gross colony photosynthesis (Fig 9B), consistent with the results shown in Fig. 7.

## Discussion

This study shows how food availability and light-intensity modulate the effects of temperature on photosynthetic capacity for symbionts within two coral species, *Stylophora pistillata* and *Turbinaria reniformis*. Contents of photosynthetic pigments (chlorophyll) and key proteins (PS2, PS1, Rubisco) were primarily affected by food availability for the branching coral species *Stylophora*, although temperature stress also caused a drop in PS2 content for unfed colonies of *Stylophora*. On the other hand, the levels of chlorophyll and key proteins in *Turbinaria* were consistently lower at high temperature, but symbionts within this species also adjusted their photosynthetic machinery according to light and feeding regimes. Contrary to the impairment of PS2 function under temperature stress, electron transport through PS1 was slightly higher, on average, at high compared with control incubation temperature. However, this increase in PS1 electron flow did not result in increased photosynthesis (measured using oxygen respirometry techniques) for coral colonies. These results suggest a switch towards cyclic electron flow at high temperatures and high light levels.

### Pigment and protein content

Coral bleaching manifests as a loss of tissue pigmentation, through a decrease in symbiont density or possibly a drop in the content of chlorophyll per symbiont [7]. The combination of increased seawater temperature and high light has previously been identified as a major factor inducing coral bleaching [7], [33], [34]. Our results confirm that availability of prey for the coral hosts can mitigate the negative impact of temperature increases on chlorophyll content, potentially by supplying sufficient energy and nutrients to maintain photosynthetic function, or to replace degraded photosynthetic complexes. Several other studies have shown the importance of coral heterotrophy as a potential mechanism for colony survival through bleaching events [28], [35]. For example, some coral species can up-regulate their feeding rates in response to bleaching to a level that is sufficient to meet daily metabolic requirements [27]. Indeed, the positive effect of feeding on chlorophyll content observed here for *Stylophora* is consistent with several other studies on both *Stylophora* [36] and *Turbinaria* [37], [38]. For zooxanthellae, temperature-induced acceleration of photoinhibition is attributable to a reduction in the rate of repair of damaged PS2 reaction centers for cultured symbionts [9], or to a mismatch between the magnitudes of the increase in repair rates compared to damage rates for symbionts in hospice [10]. The positive effects of food provision on symbiont photosynthetic capacity likely stem from improved ability to repair symbiont photodamage due to enhanced nutrient provision from the coral host to the zooxanthellae. More broadly, these findings for reef-building corals are congruent with research on plants and phytoplankton showing that nutrient supplementation protects against photooxidative damage [39], [40], and correspondingly helps to prevent loss of pigmentation.

Our results show that food provision to coral colonies affects the contents of PS1 and PS2 for coral symbionts: PS2 content for *Turbinaria* was highest in fed colonies and PS1 content for *Stylophora* was enhanced for fed colonies grown under low light. However, levels of these proteins, and of Rubisco, also depended upon temperature for *Turbinaria*. For both corals, Rubisco content was highest under the high light incubation but was not significantly higher than under the low-light unfed incubation. All three of the assayed proteins showed lower contents at high temperature for *Turbinaria*. This finding supports the consensus view that, for coral symbionts, the light-reactions of photosynthesis are impaired during temperature stress [8], [10]. However, the lower levels of Rubisco within symbionts of this coral species at high temperature, indicate an additional impairment of capacity for carbon fixation (i.e. the dark reactions of photosynthesis) [13] shown through a drop in Rubisco content [12]. For *Stylophora*, comparatively high variability among colonies within treatments meant that temperature did not significantly affect protein content. Nevertheless, colonies of this species also showed a trend toward lower content of PS2 and Rubisco at high temperature similar to that observed for *Turbinaria*. Taken in concert, these results demonstrate that temperature stress has a negative effect on multiple components of the photosynthetic machinery of coral symbionts.

As an indicator of the accuracy of the protein quantifications we compared our measurement of chl *a* content per unit coral surface area with the expected amount of chl *a* calculated from the measured levels of PS2 and PS1 proteins. Crystallographic data shows there are 35 chl molecules associated with each PS2 reaction centre [41] and 93 chl molecules associated with each PS1 reaction centre [42]. Based on the molecular weight of chlorophyll (893.5 g mol^−1^), measured levels of PsbA and PsaC (pmol µg^−1^ total protein), measured total protein content per symbiont of 3.4×10^−4^ µg symbiont^−1^ for *Turbinaria* and 2.4×10^−4^ µg symbiont^−1^ for *Stylophora* and measured symbiont densities (on the order of 10^6^ symbionts cm^−2^) we estimated that reaction centre chl *a* accounted for 24–127% of the total chl *a* pigment measured in our samples. This comparison provides an estimate of 27% as the approximate cumulative error between the measurement approaches, since reaction center chl *a* cannot exceed total chl *a*. Fed colonies of *Stylophora* maintained at low light had practically all of their chl *a* associated with reaction centres (108 – 127%). For *Turbinaria*, RC chl *a* accounted for between 24 – 80% of the total chl *a* pool and tended to be highest for fed nubbins and lowest for the colonies maintained at high light and/or exposed to high temperature, irrespective of feeding status (24 – 25%). We note that these estimates should be interpreted with caution because we did not directly observe a change in antennae versus photosystem-associated chlorophyll. However, our results are broadly consistent with a single previous study that found approximately 50% of total chl *a* present in cultured zooxanthellae was bound to the major light-harvesting complex (acpPC) and that the quantity of chl *a* associated with PS1 increased in cultures grown under low light intensity [43].

The relative contents of the different protein components of the photosynthetic machinery observed in this study varied with experimental conditions for *Turbinaria* but not for *Stylophora*. For *Turbinaria,* the ratio of Rubisco to PS2 increased following incubation under high light, as did the ratio of PS1 to PS2. Previous studies have indicated that PS1:PS2 ratios increase under conditions that favor excitation of PS2, thereby requiring additional PS1 to balance electron flow between these two reaction centres [44]. However, a recent study of physiological variation among different clades of coral symbionts showed that the ratio of functional PS1:PS2 centres (based on oxygen respirometry measurements) ranged between 0.7 to 0.2 among clades but did not vary consistently with growth irradiance [45]. In fact, among different symbiont clades there was substantial variation in the direction and magnitude of changes in this ratio with light intensity. We did not directly quantify symbiont clade identity for our experimental corals but other studies on the coral stock used in this study show that *Stylophora* harbours mainly clade A1 [46]. Presently we cannot identify the cause of these differences between coral species, or why the light dependence of the PsaC:PsbA (PS1:PS2) ratio observed here is different to that observed in another study on symbiont clade A1 [45]. Nevertheless, an increasing body of evidence points to physiological differences among clades of coral symbionts in terms of their tolerance to thermal stress [47], and their photosynthetic capacity [43], [48], [49]. We suggest that identifying the environmental correlates (light, nutrients, inorganic carbon concentration) of photoacclimation for different symbiont types, and the relative photoacclimation capacity of these groups, will be a profitable avenue of future research.

### Activity of photosystem II and photosystem I

The variability in symbiont photosynthetic activity between species, experimental conditions and temperature treatments was broadly consistent with the observed differences in PS1, PS2 and Rubisco protein contents. In this study, temperature stress caused a decrease in non-photochemical quenching capacity of PS2 for coral symbionts, whereas incubation under high light intensity increased Y(NPQ). Maximum photochemical yield was lower in colonies grown at high light intensity but high light levels also caused up-regulation of photoprotective mechanisms. Congruent with these changes, the protein assays showed that for both species RbcL (Rubisco) content was highest following incubation under high light. Moreover, for *Turbinaria*, content of PsbA (PS2) was lower under the high-light treatment, consistent with a down-regulation or impairment of PS2 function. These results support the general consensus that PS2 is the primary site of photodamage when light intensity is excessive [50]. Overall, our results regarding the function of PS2 are largely consistent with fluorometry-based studies of coral photosynthesis and photoprotection [20], [51], [52]. The novelty of our study lies in simultaneous assessment of PS1 and PS2 function and quantification of the content of key protein components of the photosynthetic machinery.

The observed divergence between rates of electron transport through PS1 and PS2 for colonies of *Turbinaria* is indicative of a change in the functional absorption cross section serving PS1 relative to that serving PS2, resulting in a change in electron flow. When the contents of PS2 versus PS1 reaction centres have been accounted for (Fig 7), then differences in excitation allocation potentially explain departures from a 1∶1 relationship between rETR(I) and rETR(II). Unfortunately, a similar analysis could not be completed for *Stylophora* because levels of PS2 were below detectible limits in the low light, fed treatment (Experiment 1, see Fig 3). Interestingly, the colonies that showed the greatest divergence from balanced electron flow through PS1 and PS2 also had a lower proportion (<40%) of their total chl *a* associated with reaction centres, suggestive of a large mobile light harvesting complex, which could shift from PS2 to PS1, increasing the light-absorption of PS1 at the expense of PS2 [53]. Such a ‘state transition’ would enhance electron flow through PS1 in a manner consistent with our results [50]. For colonies kept under low growth irradiance, rETR(I) and rETR(II) were approximately equivalent until measuring light intensity reached 131–221 µmol photons m^−2^ s^−1^ (Fig 7A,B). Above this inflection point, rETR(II) saturated but rETR(I) continued to increase, indicative of a shift to cylic electron flow above this light intensity threshold. This interpretation of our results is congruent with previous research evidencing capacity for cyclic electron transport and light-induced dissociation of antenna complexes from PS2 centres, at least for clade A *Symbiodinium,* both in culture and in *hospice* [49]. In the present study, this shift in electron flow from balanced to PS1-dominated occurred at approximately the same light level across temperature, growth light or feeding conditions; there are three data points above the inflection of each curve in each panel of Fig 7. However, despite the similarities in the overall shape of these curves, rETR(II) was substantially lower at 32°C compared to 26°C except for colonies grown under low light with food provided, showing a protective effect of feeding on temperature impairment of rETR(II). Overall these results demonstrate that the transition from linear electron flow through PS2 and PS1 towards excess rETR(I) > rETR(II), is primarily driven by light levels. Moreover, whereas the function of PS2 is impaired by temperature stress, levels of electron transport through PS1 are comparable for a given measuring light level irrespective of incubation temperature.

Cyclic electron flow, which involves only PS1 and produces ATP but not NADPH [54], is increasingly recognised as an important physiological pathway for photosynthetic and photoheterotrophic metabolism [55]. In particular, cyclic flow has been implicated in photoprotection because it generates a proton gradient that might help to dissipate excess excitation energy from PS2 [56], [57]. In cyanobacteria and plants, increased PS1 activity consistent with cyclic flow increases in cells grown under conditions with low CO_2_ availability [56], [58]. Under such conditions cyclic flow is thought to provide additional ATP required to power the carbon concentrating mechanism to enhance the availability of CO_2_ for carbon fixation [58], [59]. Coral symbionts possess Form II Rubisco [12] which has a comparatively poor ability to discriminate between CO_2_ versus O_2_ [60]. Correspondingly, ATP production through cyclic flow might serve a dual function in coral symbionts exposed to high light levels: provision of energy to drive the carbon concentrating mechanism to enhance CO_2_ availability, plus photoprotection via dissipation of excitation pressure from PS2.

### Comparison between symbiont photophysiology and colony photosynthesis

This study demonstrates that interacting physical and biological environmental factors influence how temperature stress impacts the photosynthesis of coral symbionts. Our results support the photoinhibition model of coral bleaching [7]: both species sustained chlorophyll content and photosynthesis during temperature stress when they were grown under low light intensity. Provision of nutrients, through heterotrophic feeding, was also important for maintenance of photosynthesis. Indeed, consistent with other studies, feeding mitigated the effect of temperature stress on photosynthesis [36], [61]. In general, however, the content of the major protein elements of the photosynthetic apparatus differed in their sensitivity to experimental conditions and temperature compared with total colony photosynthesis (Fig 3 compared with Fig 8). Maximum rates of photosynthesis, above light saturation, are generally limited by processes downstream of PS2 [13], [62]. Correspondingly, maximum photosynthesis rate often correlates with the concentration or activity of Rubisco [63]. In our study, colony photosynthesis was not significantly correlated with Rubisco content (Pearson's R = 0.42, p = 0.18). We are presently unable to identify the mechanism underlying this result but a likely explanation is that the activity of Rubisco might vary in response to experimental conditions and/or temperature [11], [12] independently from changes in Rubisco content. Alternatively, oxygen consumption by the coral host tissue might obscure the relationship between Rubisco content of symbionts and net oxygen evolution of coral colonies. Rates of gross photosynthesis (oxygen evolution) of colonies of *Stylophora* and *Turbinaria* were linearly correlated with electron transport through symbiont PS2 but were not significantly correlated with electron transport through symbiont PS1. Clearly, the increased electron flow through PS1 under high light did not lead to a net increase in colony oxygen evolution. This result supports our interpretation that a switch from linear to cyclic electron flow occurred at measuring light intensities above approximately 200 µmol photons m^−2^ s^−1^.

### Conclusions

Using simultaneous measurements of the function of photosystem II and photosystem I of coral symbionts, in combination with measures of total colony oxygen production and chlorophyll content and the content of three key proteins within the symbiont photosynthetic apparatus, we demonstrate that PS1 is comparatively robust to temperature stress. Our study reveals that the mechanisms regulating light harvesting and use for symbiotic dinoflagellates vary among coral species. Overall, the protein and chlorophyll content of symbionts within *Stylophora pistillata* were most strongly affected by food availability whereas these properties were strongly influenced by temperature for symbionts of *Turbinaria reniformis*. Despite these differences, a switch to increased electron flow through PS1 was apparent for both species at light intensities above approximately 200 µmol photons m^−2^ s^−1^ and the general elevation of PS1 electron transport rate at 32°C demonstrates that this photosystem was at least as active at high compared with control temperatures. Our results suggest that increased cyclic electron flow through PS1 during exposure to high temperature may act as a photoprotective mechanism for coral symbionts. In parallel, we show that provision of prey to the coral host mitigates the effects of temperature stress on symbiont photosynthesis, demonstrating that coral host metabolic status strongly influences symbiont responses to physical stresses.
